# Effectiveness of Chinese herbal medicine mouthwash in preventing ventilator-associated pneumonia in patients with endotracheal intubation: A systematic review and meta-analysis

**DOI:** 10.1097/MD.0000000000048179

**Published:** 2026-04-10

**Authors:** Yuntong Liu, Huihui Liu, Lu Ren, Shujin Yue, Zhangqi Li, Xiaoda Li, Chunxiang Su

**Affiliations:** a School of Nursing, Beijing University of Chinese Medicine, Beijing, China; b Collaborating Centre of Joanna Briggs Institute, Beijing University of Chinese Medicine, Beijing, China; c Medical and Health Analysis Center, Peking University, Beijing, China.

**Keywords:** Chinese herbal medicine mouthwash, endotracheal intubation, meta-analysis, systematic review, ventilator-associated pneumonia

## Abstract

**Background:**

To systematically evaluate the effectiveness and safety of Chinese herbal medicine mouthwash (CHMM) in preventing ventilator-associated pneumonia (VAP) in patients with endotracheal intubation (ETT).

**Methods:**

Computer searches were conducted for relevant randomized controlled trials (RCTs) in the China National Knowledge Infrastructure (CNKI), WANFANG DATA, Chinese Scientific and Technical Journal Database (VIP), Chinese Biomedical Literature Database (SinoMed), PubMed, Embase, Cochrane Library, and CINAHL Database from the establishment to April 5, 2023. The data were analyzed by RevMan 5.3 software. The methodological quality of papers was evaluated by the Cochrane version 5.1.0 and the certainty of evidence was evaluated by the GRADE system.

**Results:**

We included 27 RCTs with 2426 patients. Most of the included studies were of poor methodological quality. Meta-analysis showed that CHMM (RR = 0.41, 95% CI: 0.34–0.50, *P* < .00001, low-certainty evidence) or CHMM + routine oral care (RR = 0.31, 95% CI: 0.18–0.56, *P* < .0001) could reduce the incidence of VAP compared to non-CHMM. CHMM may be superior to saline (low-certainty evidence) and chlorhexidine (moderate-certainty evidence) in preventing VAP (*P* < .05).

**Conclusion:**

Low-certainty evidence shows that CHMM may be more advantageous in reducing the incidence of VAP compared to non-CHMM. However, due to some limitations and the low certainty of evidence, we cannot recommend CHMM for routine clinical use at this time. Large-scale and high-quality RCTs are also needed to definitively evaluate the safety and clinical benefits of CHMM in the future.

## Graphical abstract

**Figure:**
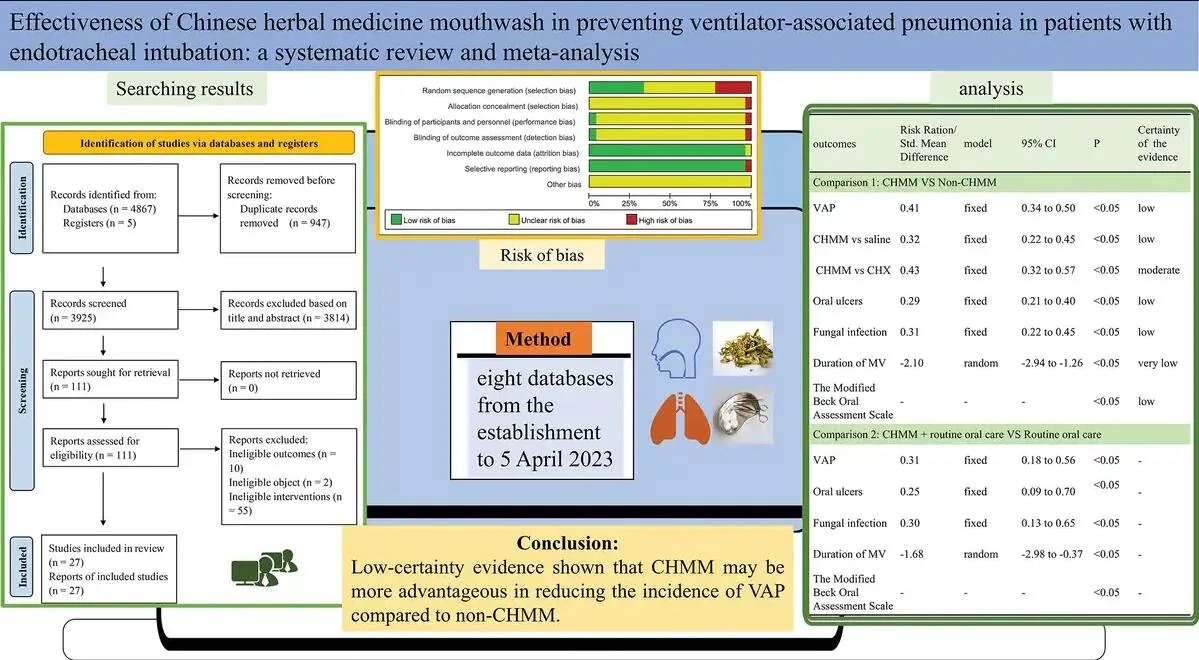

## 1. Introduction

Ventilator‐associated pneumonia (VAP) is defined as pneumonia developing in individuals who have received mechanical ventilation (MV) for at least 48 hours.^[1,2]^ It occurs 48 hours after MV and within 48 hours after extubation.^[3]^ Furthermore, it's the prevailing complication among patients on MV in intensive care units (ICUs), with a global incidence of 22.8%.^[4]^

Studies have shown that VAP can prolong the duration of MV, and increase the average length of ICU stay^[5]^ and the use of antimicrobials. Meanwhile, VAP has a substantial effect on the patient's condition and prognosis,^[6]^ and aggravates the healthcare burden on patients and society.^[7,8]^ Of all the types of MV, endotracheal intubation (ETT) is the most likely to cause VAP.^[9]^ The primary reason is that ETT disrupts the natural barrier against bacteria in the patient's oral and nasal cavities, resulting in the colonization of the oropharynx by a large number of microorganisms.^[10,11]^ Several studies^[10,12]^ supported the hypothesis that the most important mechanism for the development of VAP was the aspiration of colonized oropharyngeal secretions into the lower respiratory tract. So, reducing the number of oral microorganisms might hold the potential to prevent VAP.^[5,13]^ Therefore, the implementation of effective oral care plays a significant role in reducing the incidence of VAP.

Currently, the most commonly used oral care solution is chlorhexidine.^[10,14]^ Research has demonstrated that chlorhexidine appears to alter PH, lactate, nitrate, and nitrite concentrations in saliva.^[15]^ Hence, chlorhexidine may increase salivary acidity and decrease nitrite concentration, resulting in significant changes in the salivary microorganisms, thereby protecting the oral environment.^[15]^ Unfortunately, chlorhexidine exhibits cytotoxic activity in human cells, which can lead to tooth and filling staining.^[16]^ Meanwhile, chlorhexidine may also cause parotid gland swelling, oral soft tissue hyperpigmentation, allergic reactions, altered taste, burning sensation, oral mucosal ulcers, transient anesthesia, and sensory abnormalities.^[17]^ Consequently, researchers concentrated on mouthwashes that have the same or better effectiveness as chlorhexidine, but with fewer adverse effects.^[18,19]^

In recent years, traditional Chinese medicine (TCM) has shown great advantages in illness prevention. TCM practitioners have long acknowledged the significance of Chinese herbal medicine mouthwash (CHMM) for oral care.^[20]^ Research on the prevention of VAP by CHMM is gradually increasing.^[21,22]^ For example, Mojtahedzadeh et al^[23]^ conducted a systematic review of the effectiveness of CHMM in preventing VAP. Nevertheless, this study did not conduct a meta-analysis due to the lack of clarity of the outcome indicators. Gao et al^[24]^ carried out a network meta-analysis of the effect of different oral care solutions on VAP prevention. Of the 50 studies included, only 5 studies focused on CHMM. San et al^[25]^ conducted a meta-analysis of CHMM for the prevention of VAP. However, due to the inadequacy of the search strategy, some of the literature was missed. Also, they did not strictly follow the criteria specified in the manuscript to choose literature and failed to conduct subgroup analysis. Lastly, none of the 3 studies assessed the certainty of the evidence.

Therefore, our objective was to conduct a comprehensive assessment of the effectiveness and safety of CHMM in preventing VAP in patients with ETT, to provide evidence for clinical practice guidelines and clinical decisions.

## 2. Materials and methods

A protocol had been preregistered in the PROSPERO database (CRD42023433914). This systematic review was conducted following the Cochrane Handbook for Systematic Reviews^[26]^ and was reported according to the Preferred Reporting Items for Systematic Reviews and Meta-Analyses (PRISMA) 2020 statement.^[27]^

### 2.1. Search strategy

Two authors (LYT, LHH) systematically searched PubMed, Embase, Cochrane Library, CINAHL, China National Knowledge Infrastructure (CNKI), WANFANG DATA, Chinese Scientific and Technical Journal Database (VIP), and Chinese Biomedical Literature Database (Sinomed) from their inception to April 5, 2023. Published language was limited to English and Chinese. Moreover, snowball searching on references and citations of included studies was carried out.

The search terms included "Chinese medicine/Chinese herb," "mouthwash/oral care/oral nursing," "ventilator-associated pneumonia/mechanical ventilation/ventilator," etc. Details of each database search are given in Supplementary File 1, Supplemental Digital Content, https://links.lww.com/MD/R598.

### 2.2. Selection criteria

#### 2.2.1. Participants

Patients with ETT who required oral care, without VAP or respiratory infection at baseline.

#### 2.2.2. Intervention group

Patients who received CHMM in any form including raw material, herbal extract, the final product, containing active ingredient product, or combined with other non-CHMM for oral care.

#### 2.2.3. Control group

Patients who received routine oral care, placebo, chemical mouthwashes, other western medicine, or combined other non-CHMM. There is no limit to the method of intervention and the time of the intervention.

#### 2.2.4. Outcomes

Primary outcome: incidence of VAP.

Secondary outcomes: incidence of oral ulcers, incidence of fungal infection, duration of MV, the score of the Modified Beck Oral Assessment Scale, and adverse effects.

#### 2.2.5. Types of studies

We included randomized controlled trials (RCTs).

### 2.3. Exclusion criteria

The following studies were excluded: duplicate publication of the same study results; failure to report intervention information; full text was not available.

### 2.4. Data selection

We held a consensus meeting to develop unified retrieval strategies for each database. Two authors (LYT and LHH) searched independently according to the search strategy. The retrieved literature was imported into EndNote VX.8 (Clarivate Analytics) and duplicates were removed. Four researchers were divided into 2 groups: 2 researchers (LYT and LHH) were responsible for screening English papers, while the other 2 (RL and LZQ) were responsible for screening Chinese papers. For potentially relevant articles, the full text of the selected citations was evaluated in detail by 2 independent researchers. Any disagreement between reviewers at any stage of the study selection process was resolved through discussion or consultation with a third researcher (SCX).

### 2.5. Data extraction

Two authors (LYT and LHH) extracted data independently. Any disagreement was resolved through discussion between the 2 individuals or consultation with a third researcher (SCX). We used a predefined data extraction form to extract data, including author, year of publication, inclusion and exclusion criteria, sample size, intervention and control measures, duration of intervention and follow-up, and outcome indicators.

### 2.6. Risk of bias assessment

The quality of the RCTs selected was assessed by 2 authors (LYT and LHH) using the risk of bias tool recommended by the Cochrane Handbook V.5.1.0 (Cochrane Collaboration).^[28]^ Any disagreement was resolved through discussion or arbitration by a third researcher (SCX). Seven aspects were assessed as follows: random sequence generation, allocation concealment, blinding of participants and personnel, blinding of outcome assessment, incomplete outcome data, selective reporting, and other biases. Each domain was evaluated as “high risk” of bias, “low risk” of bias, or “uncertain risk” of bias. Publication bias was explored by funnel plots when there were more than 10 studies in an outcome.^[29]^

### 2.7. Data analysis

We used RevMan 5.3 software for data analysis. Similar comparisons with the same outcomes from different studies were combined. Subgroup analysis was conducted according to the different types of non-CHMM. Mean difference (MD) or standardized mean difference (SMD) was used as an effect indicator for continuous variables. For dichotomous variables, risk ratio (RR) or rate difference (RD) was used as an effect indicator. We calculated the overall effect and 95% confidence intervals (CI), and *P* < .05 was statistically significant. The size of heterogeneity was assessed using *I*^2^, and if (*P* > .10, *I*^2^ < 50%), a fixed-effect model was used. While *I*^2^ > 50%, a random-effects model was used. Finally, we performed subgroup analyses to explore the potential sources of heterogeneity and conducted sensitivity analyses by gradually eliminating studies.

The certainty of the evidence was evaluated using the GRADE system (GRADEpro 2020).^[30]^ We assessed the certainty of the body of evidence concerning the overall risk of bias of the included studies, the directness of the evidence, the consistency of the results, the precision of the estimates, and the risk of publication bias. We classified the certainty of the body of evidence into four categories: high, moderate, low, and very low.

## 3. Ethics

Ethical approval was not required because this study only drew from publicly available data.

## 4. Results

### 4.1. Literature screening process

**Figure 1.**
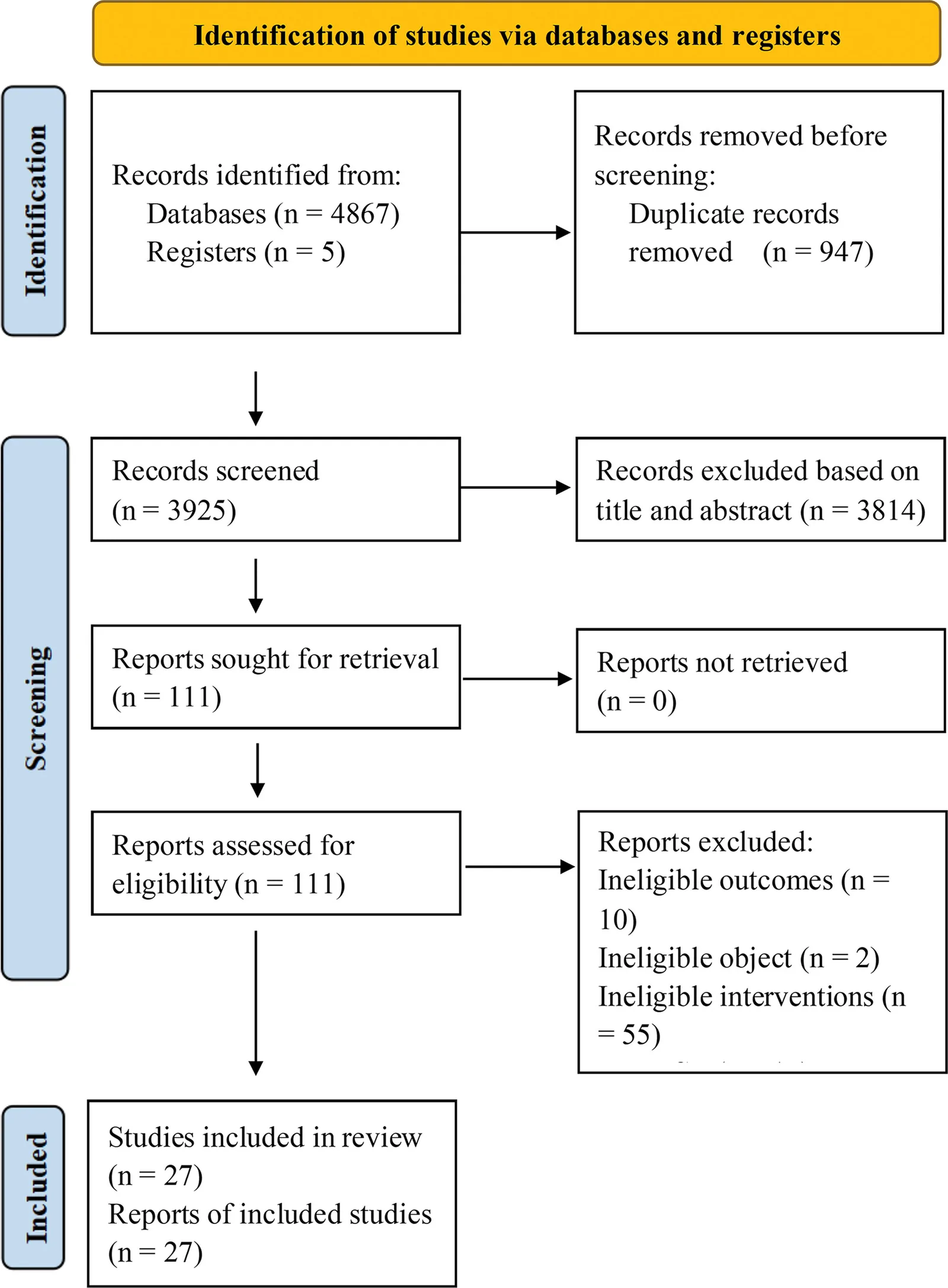
Searching results and study selection and inclusion process.

A total of 4872 documents were obtained. After eliminating duplicates and irrelevant studies, 111 full-text articles were further checked. Finally, 27 studies^[31–57]^ were included in this review (Fig. 1).

### 4.2. Basic characteristics of the studies

The majority of studies (n = 26) were undertaken in China, and the other was carried out in India. 96.3% (26/27) were published in Chinese, while 3.7% (1/27) were published in English. There were 26 trials with 2 arms and 1 trial^[31]^ with 3 arms.

We included 27 studies with a total of 2426 patients (1230 patients in the intervention group and 1196 patients in the control group). The sample size ranged from 40 to 240, with an average of 90 per trial. All studies reported inclusion and exclusion criteria. Nineteen studies^[32–36,38,40–45,47–53]^ reporting the diagnostic criteria for VAP. Eleven studies^[32,35-38,49–54]^ provided information on funding.

According to the interventions, 27 trials were classified into 2 comparisons: CHMM vs non-CHMM (n = 23); CHMM + routine oral care vs routine oral care (n = 4). Twenty-seven kinds of CHMM were investigated in the included trials, involving Chinese patent medicine (n = 2) and practitioner-prescribed herbal formula (n = 25). The frequently used herbal ingredients included *lonicerae japonicae flos*, *menthae herba*, *licorice*, *scutellariae radix*, t*araxacum* and *forsythia*. Quality standards of the herbal preparations were described in 16 trials. The frequency of oral care was 2 to 4 times per day in most studies, while the duration of oral care was 5 to 15 days. Oral care in the majority of the studies^[31,32,35,37,38,40,47,50,53]^ was performed using negative pressure rinse and cotton ball scrub. Incidence of VAP as the primary outcome was identified in all trials. Most trials described the incidence of oral ulcers and fungal infection. Only 7 trials reported the duration of MV, and 2 trials reported the score of the Modified Beck Oral Assessment Scale. However, no trial mentioned adverse effects. The main characteristics of the included studies are presented in Table 1.

**Table 1:** The basic characteristics of the 27 included studies.

Study	Sample size	Oral care solution		Methods of intervention	Duration	Ingredients of CHMM	Outcome
		Intervention	Control				
Zhao and Hua 2011^[31]^	90	Clearing heat and nourishing yin liquid (n = 30)	A: Saline (n = 30)	Negative pressure rinse with 100 mL of oral care solution, twice daily	NR	*Taraxacum, chrysanthemi indici flos, lonicerae japonicae flos, menthae herba, dendrobium, scrophulariaceae, folium perillae, adenophora stricta miq*	① ② ③
			B: CHX (n = 30)				
He et al^[32]^	60	Yinlian gargle (n = 30)	Saline (n = 30)	Negative pressure rinse and cotton ball scrub with 150–200 mL of oral care solution, 3 times daily	10 d	*Lonicerae japonicae flos, forsythia, imperatae rhizoma, rhizoma pinelliae*	① ②
Li 2014^[33]^	60	Sanhuang soup subtraction (n = 30)	Saline (n = 30)	Negative pressure rinse with 300 mL of oral care solution, twice daily	NR	*Coptidis rhizoma, scutellariae radix, phellodendri chinrnsis cortex, caulis bambusae in tenia, houttuyniae herba, citrus reticulata, platycodon grandiforus, bamusaeconcretiosilicea, belamcandae rhizome, dried phragmitis rhizoma root, panax notoginseng, menthae herba, licorice*	① ②
Lin et al 2014^[34]^	139	Boron powder (n = 67)	2% hydrogen peroxide (n = 72)	Routine oral care with 30 mL oral care solution, 4 times daily	NR	*Borneolum syntheticum, borax, saposhnikoviae radix, licorice, lonicerae japonicae flos, forsythia, menthae herba, schizonepetae herba, angelica dahurica, coptidis rhizoma, phellodendri chinrnsis cortex*	①
Wu et al 2016^[35]^	60	Golden jade mouthwash (n = 34)	Saline (n = 26)	Negative pressure rinse and cotton ball scrub with 30–40 mL oral care solution, 3 times daily	7 d	*Lonicerae japonicae flos, menthae herba, polygonati odorati rhizoma, licorice*	① ②
Xu et al^[36]^	50	Golden jade mouthwash (n = 25)	0.3% hydrogen peroxide (n = 25)	Negative pressure rinse and toothbrush with 50 mL oral care solution, 3 times daily	NR	*Lonicerae japonicae flos, menthae herba, polygonati odorati rhizoma, licorice*	① ②
Zhang et al 2016^[37]^	68	CHMM (n = 34)	Saline + CHX (n = 34)	Negative pressure rinse and cotton ball scrub with 50 mL oral care solution, 4 times daily	NR	*Lonicerae japonicae flos, ophiopogon japonicus, menthae herba, licorice, isatis tinctoria L., taraxacum, pogostemon cablin, forsythia*	① ② ③
Dong et al2017^[38]^	40	Double-flower collutory (n = 20)	Saline (n = 20)	Negative pressure rinse and cotton ball scrub with 300 mL oral care solution	8 days	*Lonicerae japonicae flos, borax, licorice*	①
Li et al 2017^[39]^	78	Chinese herbal rushes gargle (n = 39)	Saline (n = 39)	Negative pressure rinse and cotton ball scrub with 500 mL oral care solution, 3 times daily	NR	*Phragmitis rhizoma, menthae herba, folium perillae*	① ② ③
Shi and Huang 2017^[40]^	82	Routine oral care + CHMM (n = 41)	Routine oral care (n = 41)	Negative pressure rinse with oral care solution, twice daily	15 d	*Licorice, lonicerae japonicae flos, menthae herba*	① ② ③
Li et al 2018^[41]^	60	Yinqi kushen decoction (n = 30)	Saline (n = 30)	Negative pressure rinse and cotton ball scrub with 100 mL oral care solution, 4 times daily	NR	*Lonicerae japonicae flos, hedysarum multijugum maxim., sophorae flavescentis radix*	①
Gao 2019^[42]^	100	CHMM (n = 50)	Compound CHX (n = 50)	Negative pressure rinse and cotton ball scrub with 30 mL oral care solution, 3 times daily	NR	*Scutellariae radix/gypsum, licorice, anemarrhenae rhizoma, japonica rice*	① ②
Li et al 2019^[43]^	120	Sanhuang soup (n = 60)	Compound CHX (n = 60)	Negative pressure rinse and cotton ball scrub with 50–100 mL oral care solution, 4 times daily	10 d	*Coptidis rhizoma, scutellariae radix, phellodendri chinrnsis cortex, caulis bambusae in tenia, stemonae radix, houttuyniae herba, citrus reticulata, platycodon grandiforus, bamusaeconcretiosilicea, dried phragmitis rhizoma root, belamcandae rhizome, menthae herba, panax notoginseng, licorice*	① ②
Yang et al 2019^[44]^	80	Compound tea polyphenol gargle (n = 40)	Saline (n = 40)	Routine oral care with oral care solution, 4 times daily	NR	Compound tea polyphenols	①
Gao 2020^[45]^	120	White tiger soup (n = 60)	0.12% CHX (n = 60)	Routine oral care with oral care solution	NR	*Gypsum, licorice, anemarrhenae rhizoma, japonica rice*	①
Liang and Zhao 2020^[46]^	164	CHMM (n = 82)	Saline (n = 82)	Negative pressure rinse and toothbrush, twice daily	7 d	*Lonicerae japonicae flos, scutellariae radix, menthae herba, licorice, eupatorium fortunei turcz, clove*	① ② ④ ⑤
Wang et al 2020^[47]^	60	Routine oral care + CHMM (n = 30)	Routine oral care (n = 30)	Negative pressure rinse and toothbrush with 100 mL oral care solution, twice to 3 times daily	5 d	*Eupatorium fortunei turcz, lonicerae japonicae flos, menthae herba, licorice*	① ④ ⑤
Fu and Xia 2021^[48]^	100	Shuanghua yin (n = 50)	CHX (n = 50)	Negative pressure rinse and cotton ball scrub with 500 mL oral care solution, 3 times daily	Until off ventilator	*Chrysanthemum, lonicerae japonicae flos, taraxacum, pogostemon cablin, menthae herba, forsythia, scutellariae radix*	①② ③ ④
Xu 2021^[55]^	100	CHMM (n = 50)	0.1% povidone-iodine (n = 50)	Negative pressure rinse and toothbrush with oral care solution, 3 times daily	NR	*Lonicerae japonicae flos, phragmitis rhizoma, menthae herba, amomum aurantiacum, lablab semen album*	①
Yu et al 2021^[56]^	50	CHMM + routine oral care (n = 25)	Routine oral care (n = 25)	Negative pressure rinse and cotton ball scrub with 100 mL oral care solution, 3 times daily	10 d	*Lonicerae japonicae flos, chrysanthemum*	①
Zhang et al 2021^[53]^	240	Jindan mouthwash (n = 120)	Saline (n = 120)	Negative pressure rinse and cotton ball scrub with 100 mL oral care solution, twice daily	7 d	*Lonicerae japonicae flos, forsythia fructus, taraxacum, cortex moutan, licorice*	① ③
Liao et al 2022^[49]^	60	Sanhuang diarrhea heart soup (n = 30)	0.12% CHX (n = 30)	Negative pressure rinse and cotton ball scrub with 100 mL oral care solution, twice daily	Until 24 h after extubation	*Radix rhei et rhizome, coptis rhizoma, scutellariae radix*	① ②
Lin et al 2022^[50]^	60	Pujiang gargarism (n = 30)	Compound CHX (n =30)	Negative pressure rinse and toothbrush with 100mL, 4 times daily	7 d	*Taraxacum, pollen typhae, zingiberis rhizoma, psoralea corylifolia, borneolum syntheticum*	① ② ③ ④
Wu et al 2022 ^[51]^	80	Self-developed heat-clearing and toxin-removing soup + routine oral care (n = 39)	Routine oral care (n = 38)	Negative pressure rinse and cotton ball scrub with 200 mL oral care solution, 3 times daily	14 d	*Cinnamomi ramulus, licorice, scutellariae radix, paris, bletilla striata, rhizoma paridis, menthae herba*	① ② ③ ④
Xie et al 2022^[52]^	80	Clearing heat and dampness-removing liquid (n = 40)	0.12% CHX (n = 40)	Negative pressure rinse and toothbrush with 100 mL oral care solution, 4 times daily	15 d	*Scutellariae radix, bletilla striata, caryophylliflos, aucklandiae radix, cimicifugae rhizoma, fructus arctii, pollen typhae, gardeniae fructus, sophorae flavescentis radix*	① ② ③ ④
Feng et al 2023^[54]^	60	Self-developed CHMM (n = 30)	Compound CHX (n = 30)	Routine oral care with 100 mL oral care solution, twice to 3 times daily	14 d	*Lonicerae japonicae flos, lithospermum erythrorhizon, chrysanthemi indici flos, eriobotryae folium, menthae herba*	① ② ③ ④
Jahanshir et al 2023^[57]^	168	6.66% clove mouthwash (n = 84)	0.2% CHX (n = 84)	Negative pressure rinse and cotton ball scrub with 15 mL oral care solution, twice daily	5 d	*Clove* extract	①

Explanations.

① Incidence of VAP, ② incidence of oral ulcers, ③ incidence of fungal infections, ④ duration of MV, ⑤ the score of the Modified Beck Oral Assessment Scale, ⑥ adverse effects.

CHMM = Chinese herbal medicine mouthwash, CHX = Chlorhexidine, NR = Not reported.

### 4.3. Risk of bias in included studies

**Figure 2.**
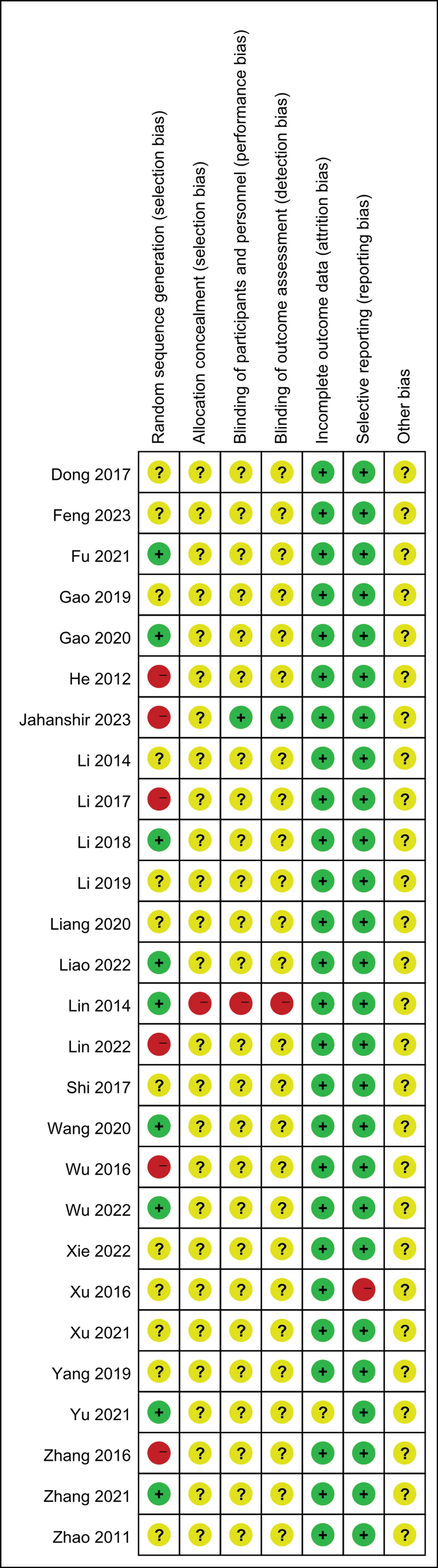
Risk of bias summary graph.

Fifteen studies^[32,34,35,37,39,41,45,47–51,53,56,57]^ reported the method of random sequence generation. Random number table was used in 9 studies,^[34,41,45,47–49,51,53,56]^ and randomization was based on the order of hospitalization or consultation in the other 6 studies.^[32,35,37,39,50,57]^ None of the studies mentioned the method of allocation concealment. One study^[57]^ was a comparative randomized triple-blind clinical trial. One^[34]^ clearly stated that the study was an open clinical trial. One study^[56]^ did not mention the problem of sample shedding. One study^[36]^ failed to report all the outcomes specified in the methods section. The calculation of sample size was conducted in only 1 study.^[57]^ The specifics of the risk of bias for the included studies are shown in Figure 2.

### 4.4. Meta-analysis results

#### 4.4.1. Comparison 1: CHMM VS Non-CHMM

##### 4.4.1.1. Incidence of VAP

**Figure 3.**
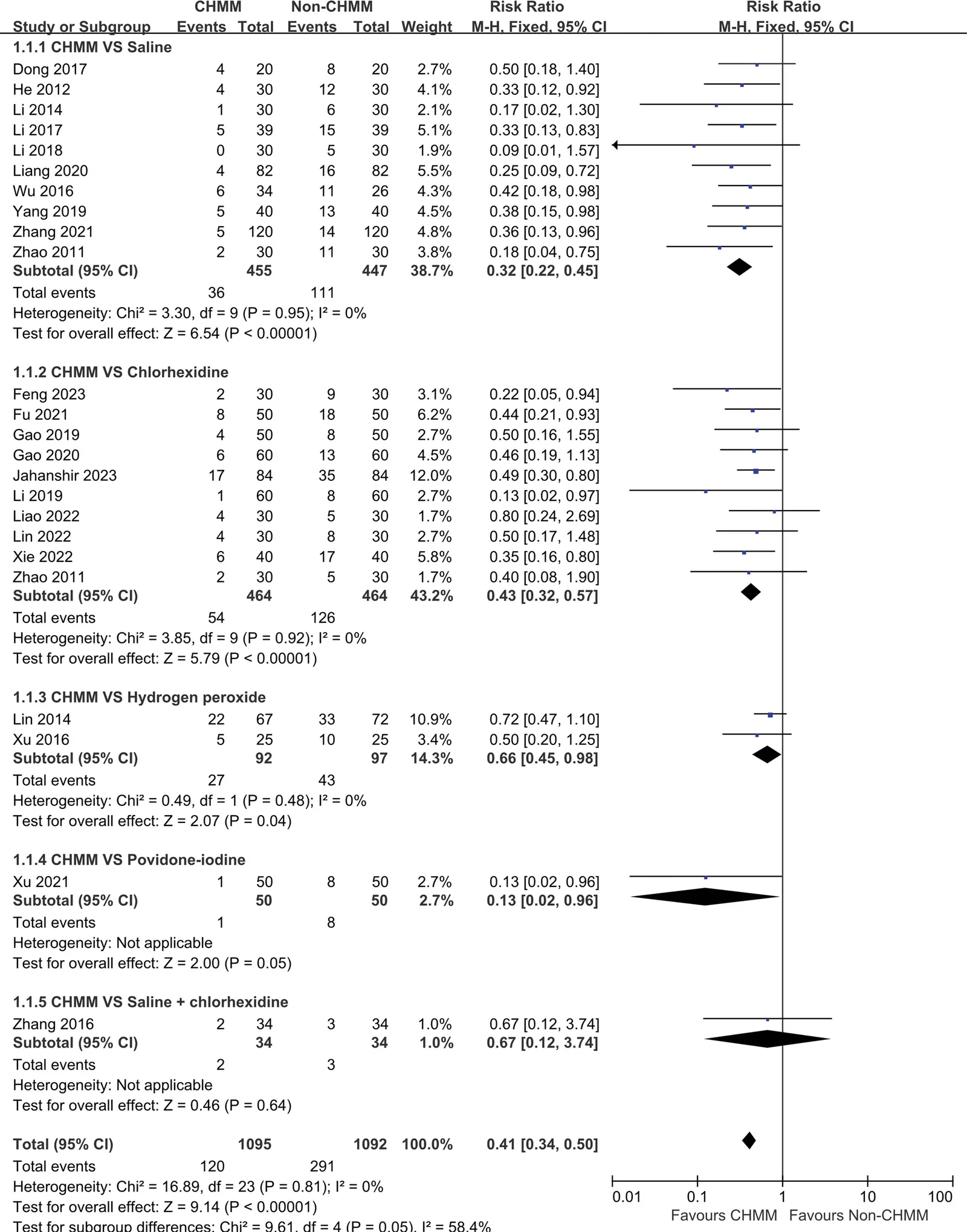
Forest plot of incidence of VAP in CHMM vs non-CHMM. CHMM = Chinese herbal medicine mouthwash, VAP = ventilator-associated pneumonia.

**Figure 4.**
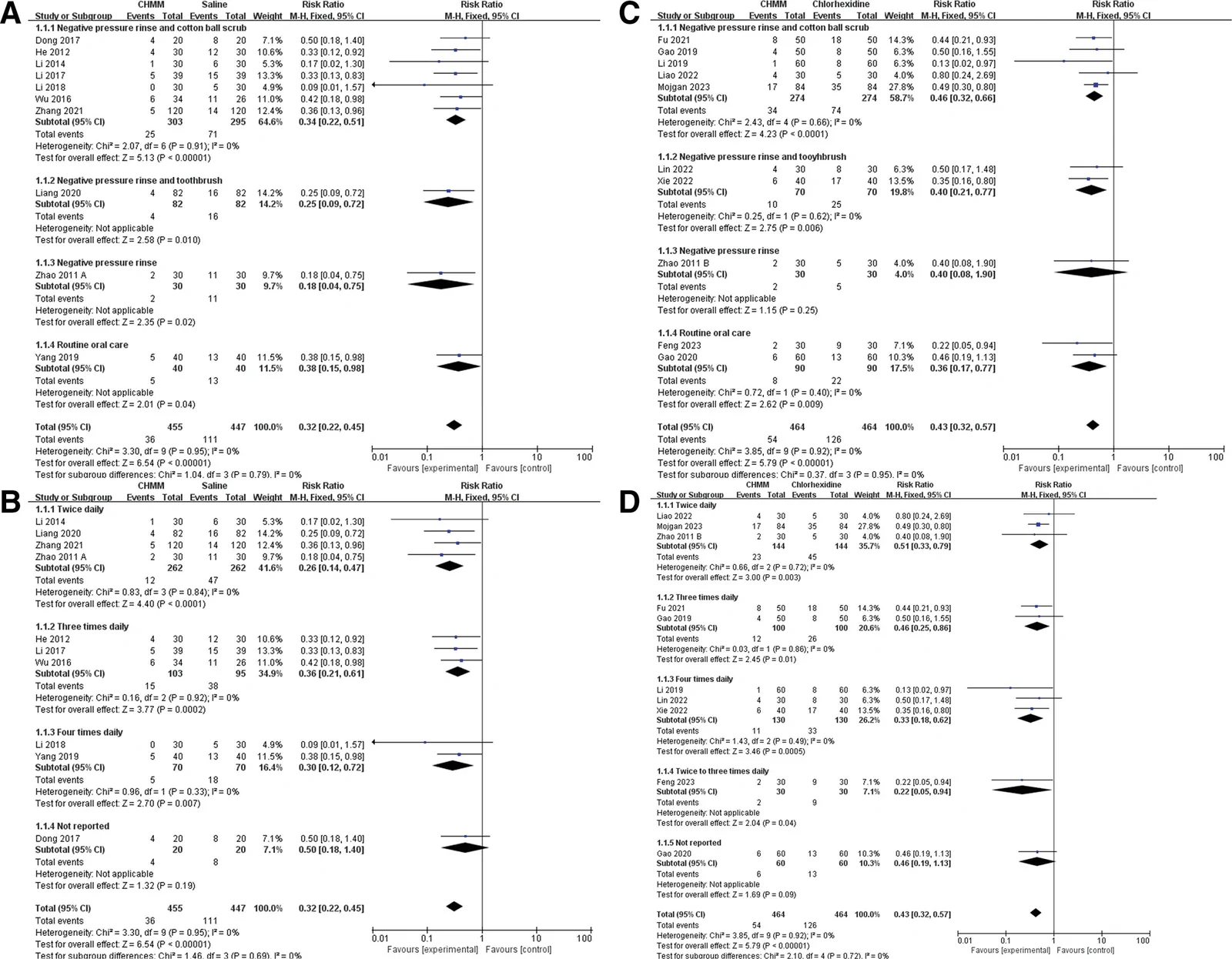
Forest plot of incidence of VAP in subgroup analyses by control type, care frequency and method. (A) Forest plot of incidence of VAP in Subgroup analysis by oral care methods: CHMM versus Saline. (B) Forest plot of incidence of VAP in Subgroup analysis by oral care frequency: CHMM versus saline. (C) Forest plot of incidence of VAP in Subgroup analysis by oral care methods: CHMM versus chlorhexidine. (D) Forest plot of incidence of VAP in Subgroup analysis by oral care frequency: CHMM versus chlorhexidine. CHMM = Chinese herbal medicine mouthwash, CI = confidence interval, VAP = ventilator-associated pneumonia.

**Figure 5.**
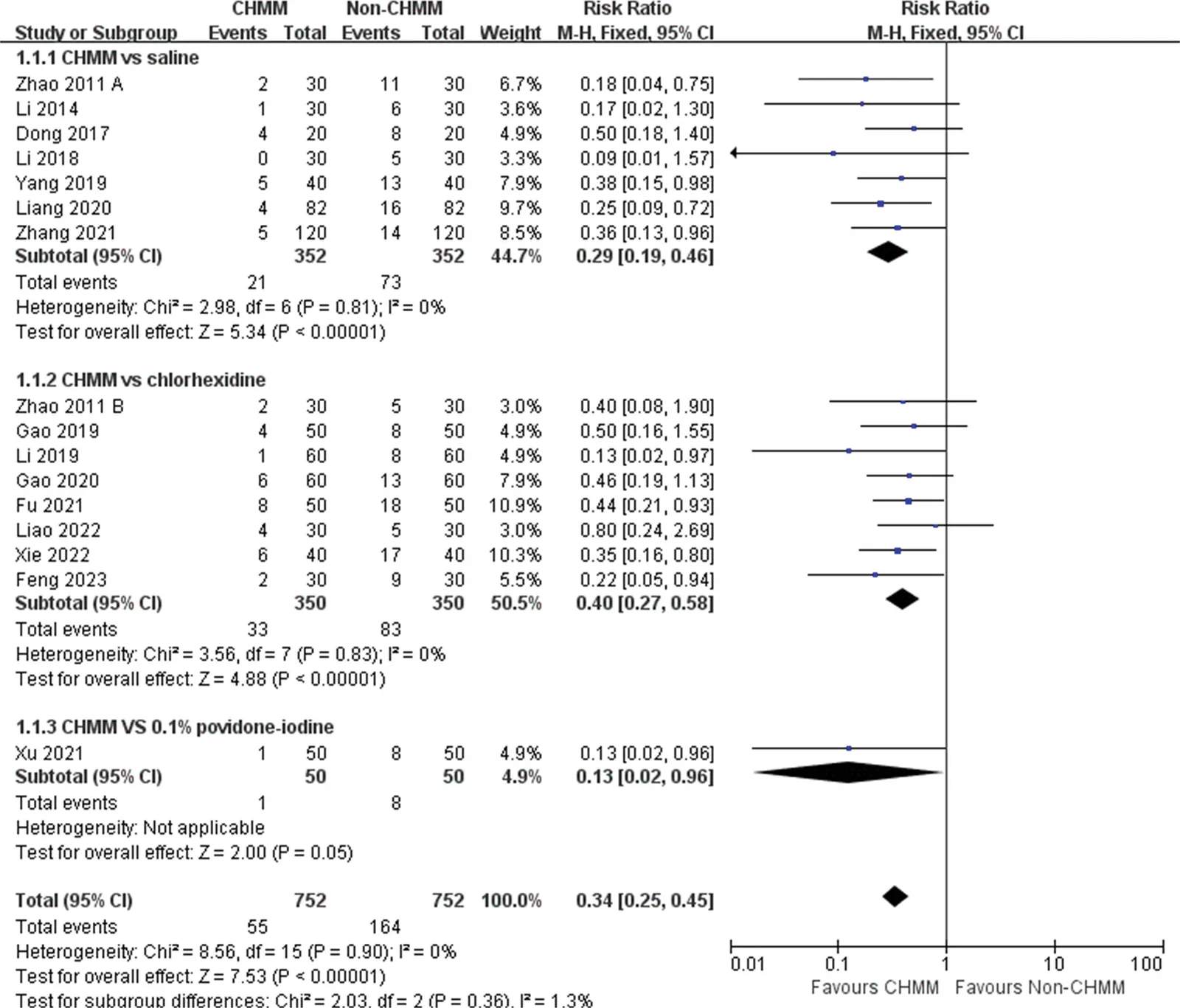
Forest plot of incidence of VAP in CHMM vs non-CHMM restricted to studies with a low risk of bias. CHMM = Chinese herbal medicine mouthwash, CI = confidence interval, VAP = ventilator-associated pneumonia.

A total of 23 studies^[37–39,41–46,48–50,52–55,57]^ reported the incidence of VAP. Overall, the meta-analysis of 23 studies using any type of non-CHMM showed that CHMM probably reduced the incidence of VAP (*P* = .81, *I*^2^ = 0%, fixed-effect model, RR = 0.41, *P* < .00001, 95% CI: 0.34–0.50, low-certainty evidence). The results of the sensitivity analysis had not changed substantially, we would have regarded our conclusions as stable with a higher degree of certainty. Among these, ten studies^[31–33,35,38,39,41,44,46,53]^ (902 participants) compared CHMM with saline. The meta-analysis showed that the effectiveness of CHMM in preventing VAP was probably better than saline (*P* = .95, *I*^2^ = 0%, fixed-effect model, RR = 0.32, 95% CI: 0.22–0.45, *P* < .00001, low-certainty evidence). Furthermore, subgroup analyses stratified by oral care methods and the daily frequency of oral care yielded consistent results. Ten studies^[31,42,43,45,48–50,52,54,57]^ (928 participants) have found that CHMM may have potentially greater efficacy than chlorhexidine rinse in reducing the incidence of VAP (*P* = .92, *I*^2^ = 0%, fixed-effect model, RR = 0.43, *P* < .00001, 95% CI: 0.32–0.57, moderate-certainty evidence). Besides, subgroup analyses stratified by oral care methods and daily frequency of oral care did not alter this conclusion. Two studies comparing CHMM to hydrogen peroxide^[34,36]^ (189 participants) demonstrated a significant decrease in the incidence of VAP in the CHMM group (*P* = .48, *I*^2^ = 0%, fixed-effect model, RR = 0.66, *P* = .04, 95% CI: 0.45–0.98). Another study^[55]^ (100 participants) showed that CHMM did not reduce the incidence of VAP when compared to 0.1% povidone-iodine (*P* = .05). Zhang’s study^[37]^ compared CHMM with saline + chlorhexidine, and there was no significant difference in the incidence of VAP (*P* = .64; see Figs 3 and 4). Lastly, we conducted a sensitivity analysis restricted to studies with a low risk of bias. The result demonstrated no material difference from the primary analysis (RR = 0.34, *P* < .00001, 95% CI: 0.25–0.45; Fig. 5).

##### 4.4.1.2. Incidence of oral ulcers

**Figure 6.**
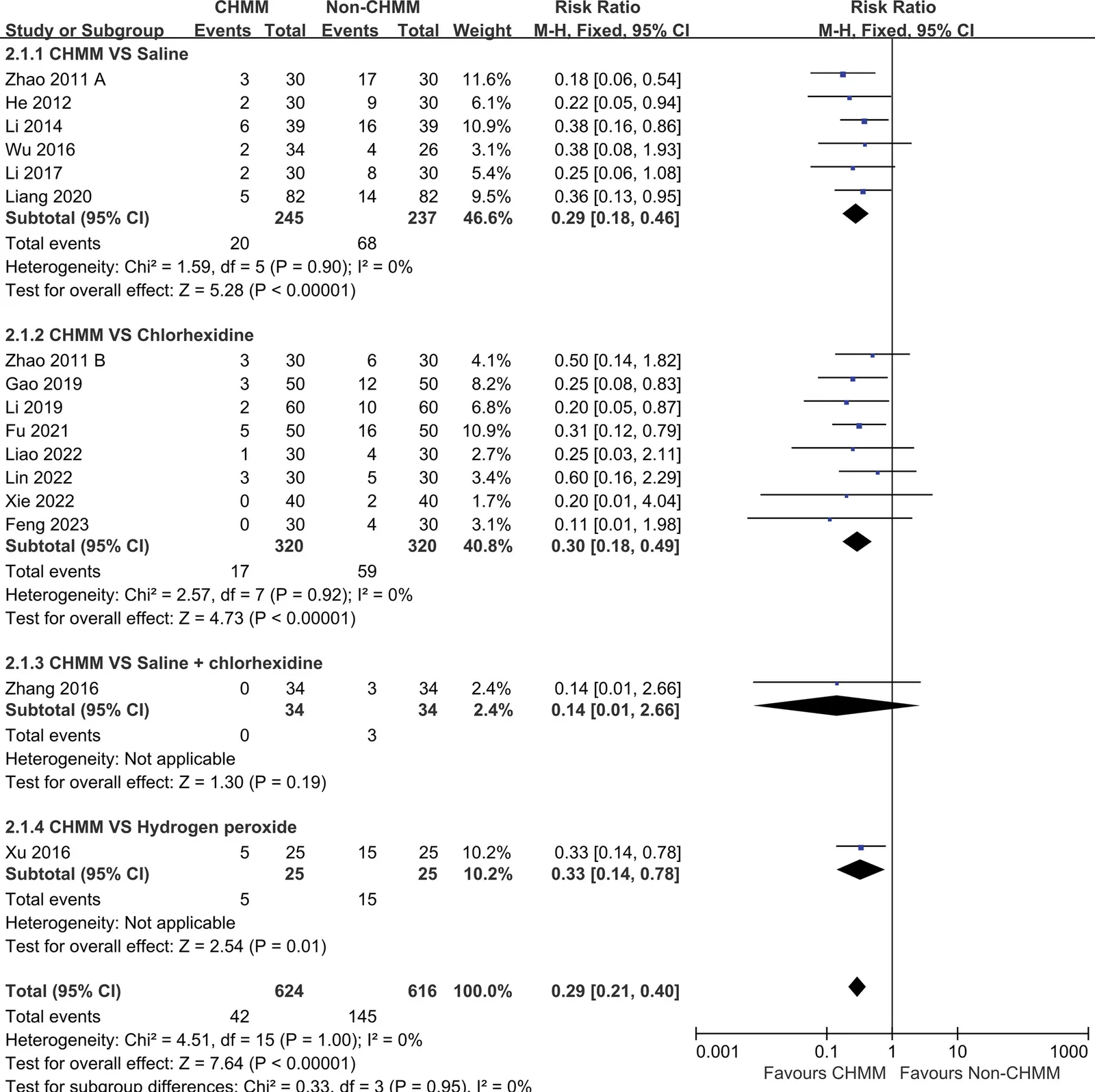
Forest plot of incidence of oral ulcers in CHMM vs non-CHMM. CHMM = Chinese herbal medicine mouthwash, CI = confidence interval.

Fifteen studies^[31–33,35–37,40,42,43,46,48–50,52,54]^ contributed results to this outcome. Overall, the meta-analysis of 15 studies using any type of non-CHMM showed that CHMM probably reduced the incidence of oral ulcers (*P* = 1.00, *I*^2^ = 0%, fixed-effect model, RR = 0.29, *P* < .00001, 95% CI: 0.21–0.40, low-certainty evidence). The result of the sensitivity analyses did affect the overall result and conclusions. Among these, 6 studies^[31-33,35,39,46]^ (482 participants) compared CHMM with saline. The meta-analysis showed a reduction in the incidence of oral ulcers in the CHMM group (*P* = .90, *I*^2^ = 0%, fixed-effect model, RR = 0.29, *P* < .00001 95% CI: 0.18–0.46). Eight studies^[31,42,43,48–50,52,54]^ (640 participants) compared CHMM to chlorhexidine, and the results showed that CHMM was more effective, probably in preventing oral ulcers than chlorhexidine (*P* = .92, *I*^2^ = 0%, fixed-effect model, RR = 0.30, *P* < .00001, 95% CI: 0.18–0.49). One study^[37]^ comparing CHMM to saline + chlorhexidine showed no significant difference in the incidence of oral ulcers (*P* = .19 > .05). One study^[36]^ compared CHMM with 0.3% hydrogen peroxide. The results showed that the difference was statistically significant (*P* = .01; see Fig. 6).

##### 4.4.1.3. Incidence of fungal infection

Eight studies^[31,37,39,48,50,52–54^ reported this outcome. Overall, the meta-analysis of 8 studies using any type of non-CHMM showed that CHMM probably reduced the incidence of fungal infection (*P* = .88, *I*^2^ = 0%, fixed-effect model, RR = 0.31, *P* < .00001, 95% CI: 0.22–0.45, low-certainty evidence).

**Figure 7.**
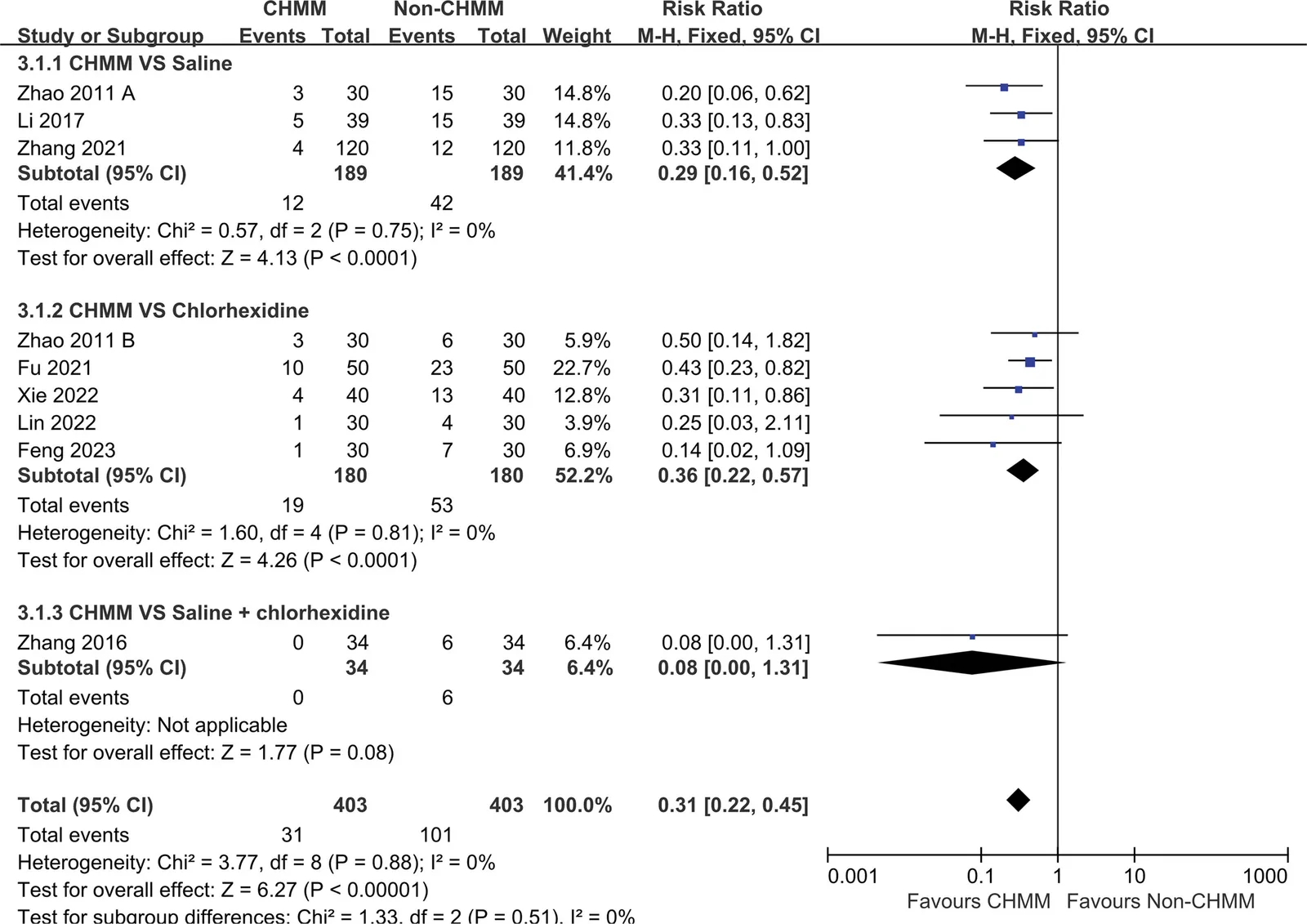
Forest plot of incidence of fungal infection in CHMM vs non-CHMM. CHMM = Chinese herbal medicine mouthwash, CI = confidence interval.

Three studies^[31,39,53]^ (378 participants) compared CHMM with saline and showed CHMM was more effective probably in preventing fungal infections (*P* = .75, *I*^2^ = 0%, fixed-effect model, RR = 0.29, *P* < .0001, 95% CI: 0.16–0.52). Five studies^[31,48,50,52,54]^ (360 participants) compared CHMM with chlorhexidine and showed significant differences favoring CHMM in preventing fungal infection (*P* = .81, *I*^2^ = 0%, fixed-effect model, RR = 0.36, *P* < .0001, 95% CI: 0.22–0.57). One study^[37]^ demonstrated that CHMM wasn’t superior to saline + chlorhexidine (*P* = .08 > .05; see Fig. 7).

##### 4.4.1.4. Duration of MV

The duration of MV was analyzed in 5 studies^[46,48,50,52,54]^ (464 participants). Overall, the result of a meta-analysis of 5 studies using any type of non-CHMM showed that CHMM probably shortened the duration of MV (*P* < .00001, *I*^2^ = 92%, random-effect model, SMD = −2.10, *P* < .00001, 95% CI: −2.94 to −1.26, very low-certainty evidence). Sensitivity analysis of the studies was performed by excluding each study 1 by 1, and the results were unchanged.

**Figure 8.**
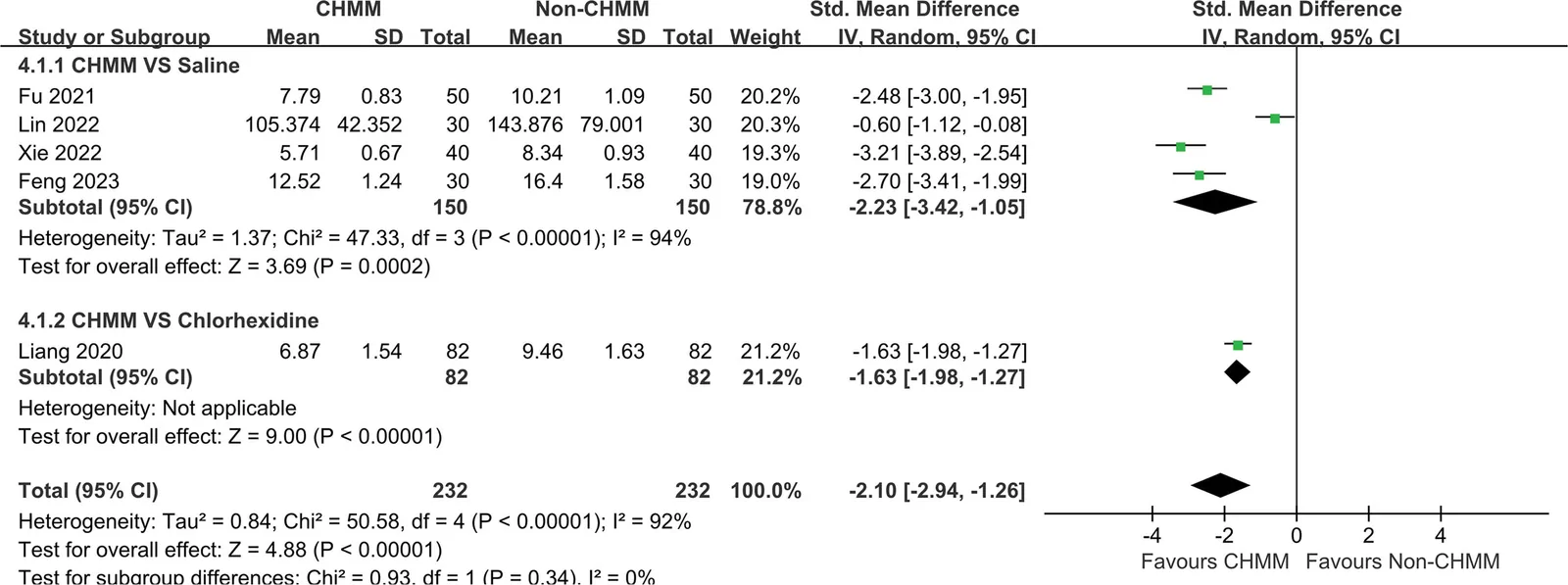
Forest plot of duration of MV in CHMM vs non-CHMM. CHMM = Chinese herbal medicine mouthwash, CI = confidence interval, MV= mechanical ventilation, SD = standard deviation.

The results of the meta-analysis illustrated that the duration of MV was shorter in patients with CHMM than saline (*P* < .00001, *I*^2^ = 94%, random-effect model, SMD = −2.23, *P* = .0002, 95% CI: −3.42 to −1.05). Sensitivity analysis of the studies was performed by excluding each study 1 by 1, and the results were unchanged. The other study^[46]^ indicated that CHMM may be superior to chlorhexidine in shortening the duration of MV (*P* < .00001; see Fig. 8).

##### 4.4.1.5. The score of the Modified Beck Oral Assessment Scale

Only 1 study^[46]^ reported this outcome. The result showed that the score of the Modified Beck Oral Assessment Scale in the CHMM group was lower than the control group (*P* < .05, low-certainty evidence).

##### 4.4.1.6. Adverse effects

None of the studies reported this outcome.

#### 4.4.2. Comparison 2: CHMM + routine oral care vs routine oral care

##### 4.4.2.1. Incidence of VAP

**Figure 9.**
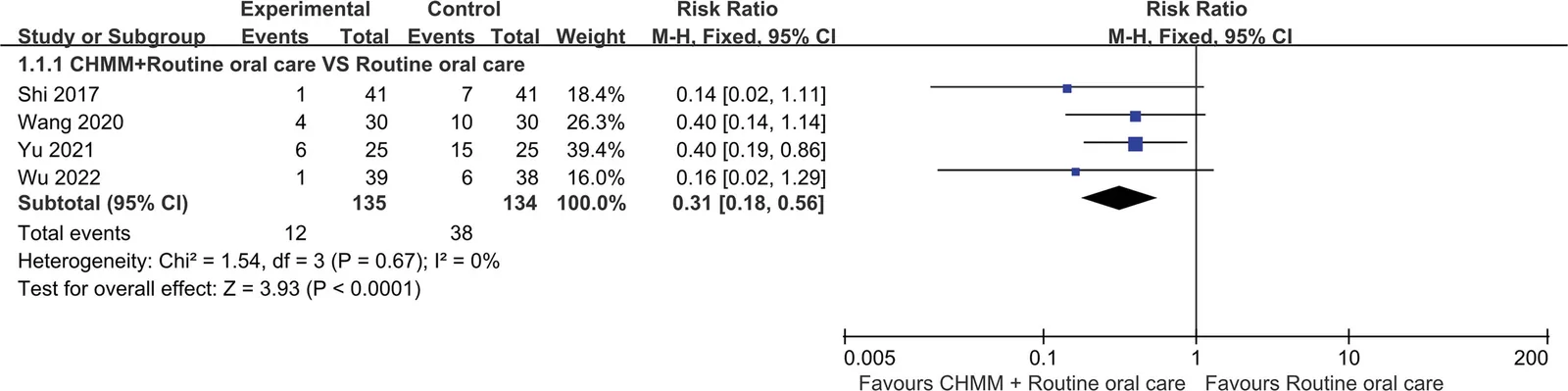
Forest plot of incidence of VAP in CHMM + routine oral care vs routine oral care. CHMM = Chinese herbal medicine mouthwash, CI = confidence interval, VAP = ventilator-associated pneumonia

Four studies^[40,47,51,56]^ (269 participants) provided data on this outcome. Overall, the meta-analysis of 4 studies showed that CHMM + routine oral care reduced the incidence of VAP (*P* = .67, *I*^2^ = 0%, fixed-effect model, RR = 0.31, *P* < .0001, 95% CI: 0.18–0.56). For the primary outcome, we conducted a sensitivity analysis. The results had not changed substantially in sensitivity analyses. We would have regarded our conclusions as stable with a higher degree of certainty (see Fig. 9).

##### 4.4.2.2. Incidence of oral ulcers

**Figure 10.**
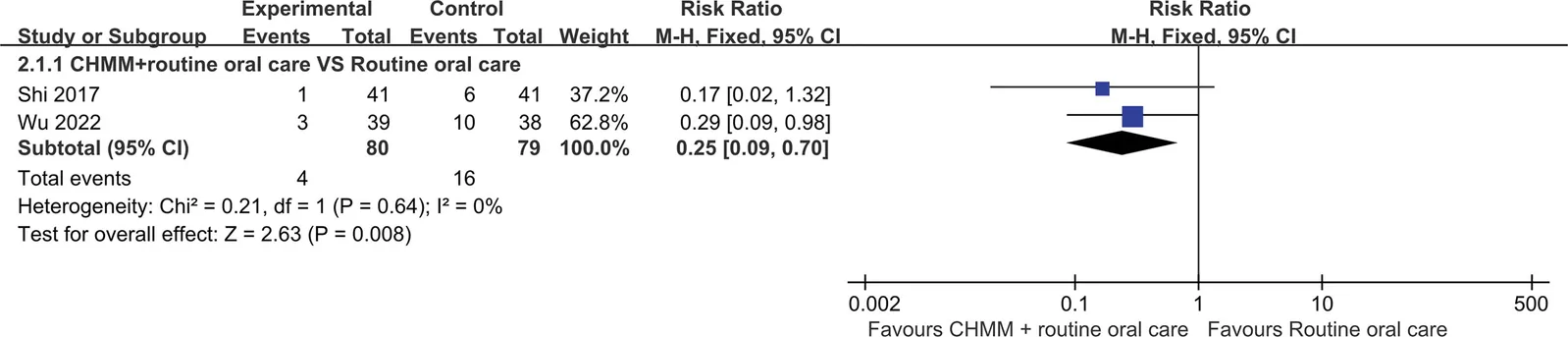
Forest plot of incidence of oral ulcers in CHMM + routine oral care vs routine oral care. CHMM = Chinese herbal medicine mouthwash, CI = confidence interval.

Two studies^[40,51]^ (159 participants) contributed results to this outcome and demonstrated that CHMM + non-CHMM probably reduced the incidence of oral ulcers (*P* = .64, *I*^2^ = 0%, fixed-effect model, RR = 0.25, *P* = .008, 95% CI: 0.09–0.70; see Fig. 10).

##### 4.4.2.3. Incidence of fungal infection

**Figure 11.**
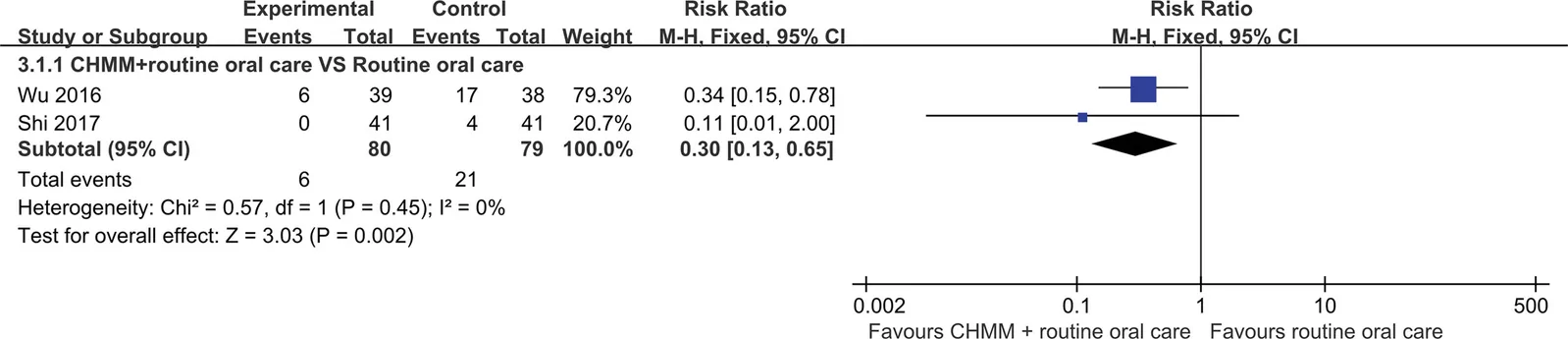
Forest plot of incidence of fungal infection in CHMM + routine oral care vs routine oral care. CHMM = Chinese herbal medicine mouthwash, CI = confidence interval.

Two studies^[35,39]^ (159 participants) reported this outcome. The results showed that CHMM + routine oral care was better than routine oral care (*P* = .45, *I*^2^ = 0%, fixed-effect model, RR = 0.30, *P* = .002, 95% CI: 0.13–0.65; see Fig. 11).

##### 4.4.2.4. Duration of MV

**Figure 12.**
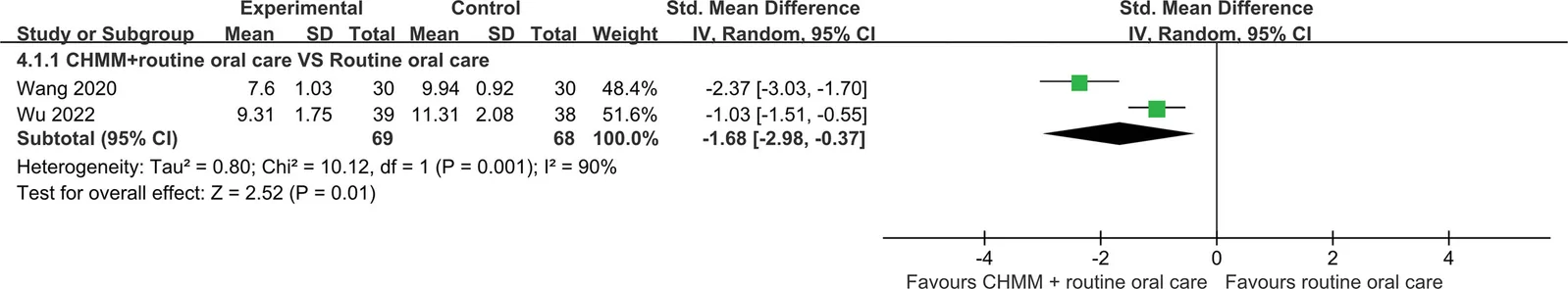
Forest plot of duration of MV in CHMM + routine oral care vs routine oral care. CHMM = Chinese herbal medicine mouthwash, CI = confidence interval, MV= mechanical ventilation, SD = standard deviation.

This outcome was demonstrated in 2 studies^[47,51]^ (137 participants), and found that the CHMM + routine oral care may shorten the duration of MV than routine oral care (*P* = .001, *I*^2^ = 90%, random-effect model, SMD = −1.68, *P* = .01, 95% CI: −2.98 to −0.37). Sensitivity analysis of the studies was performed by excluding each study 1 by 1, and the results were unchanged (see Fig. 12).

##### 4.4.2.5. The score of the Modified Beck Oral Assessment Scale

Only 1 study^[47]^ reported the outcome and demonstrated that CHMM + routine oral care may improve the oral condition and reduce the score of the Modified Beck Oral Assessment Scale when compared to routine oral care (*P* < .05).

##### 4.4.2.6. Adverse effects

None of the studies reported this outcome.

### 4.5. Risk of publication bias

**Figure 13.**
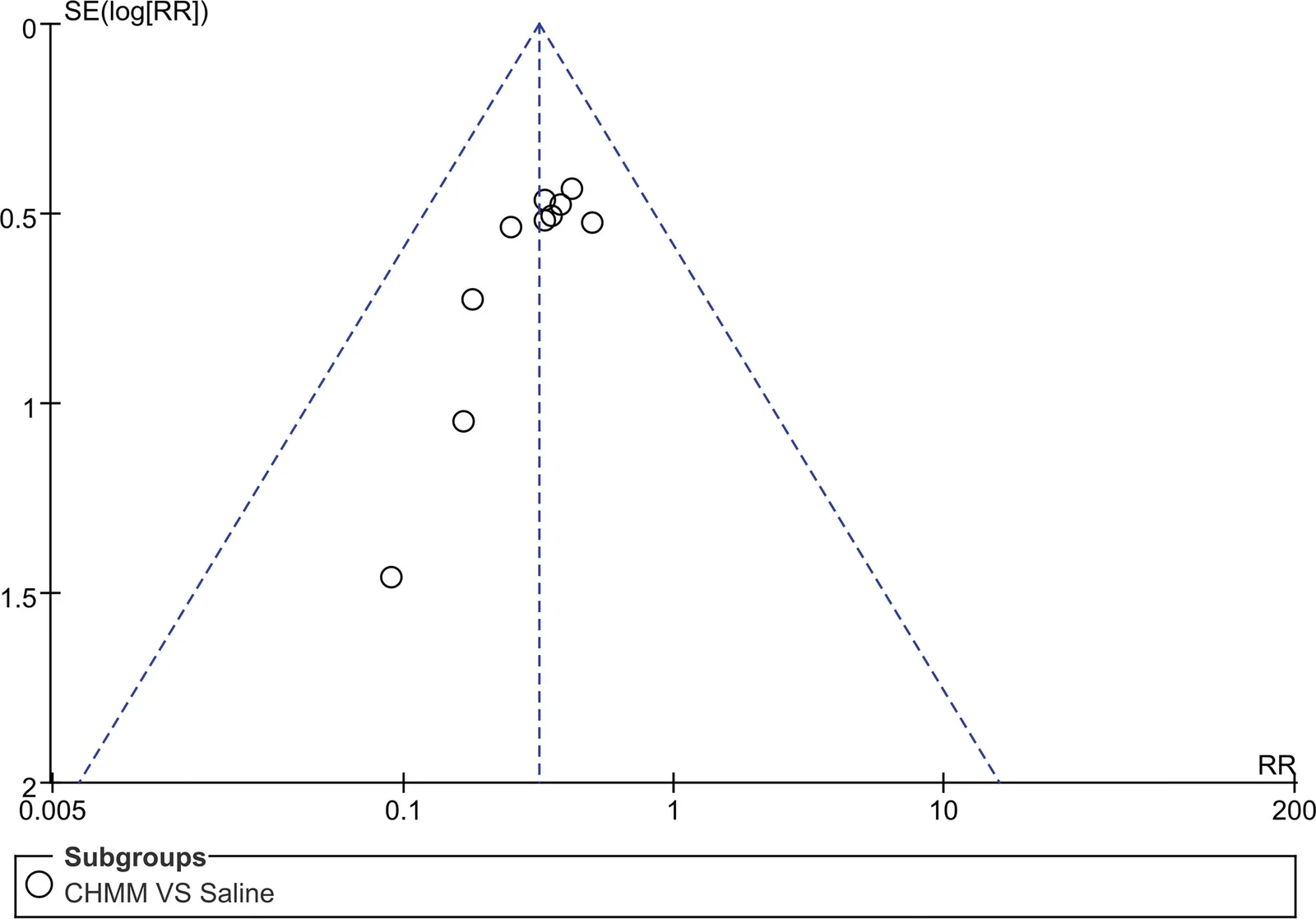
Funnel plot of the incidence of VAP in CHMM vs saline. CHMM = Chinese herbal medicine mouthwash, RR = risk ratio, SE = standard error, VAP = ventilator-associated pneumonia.

**Figure 14.**
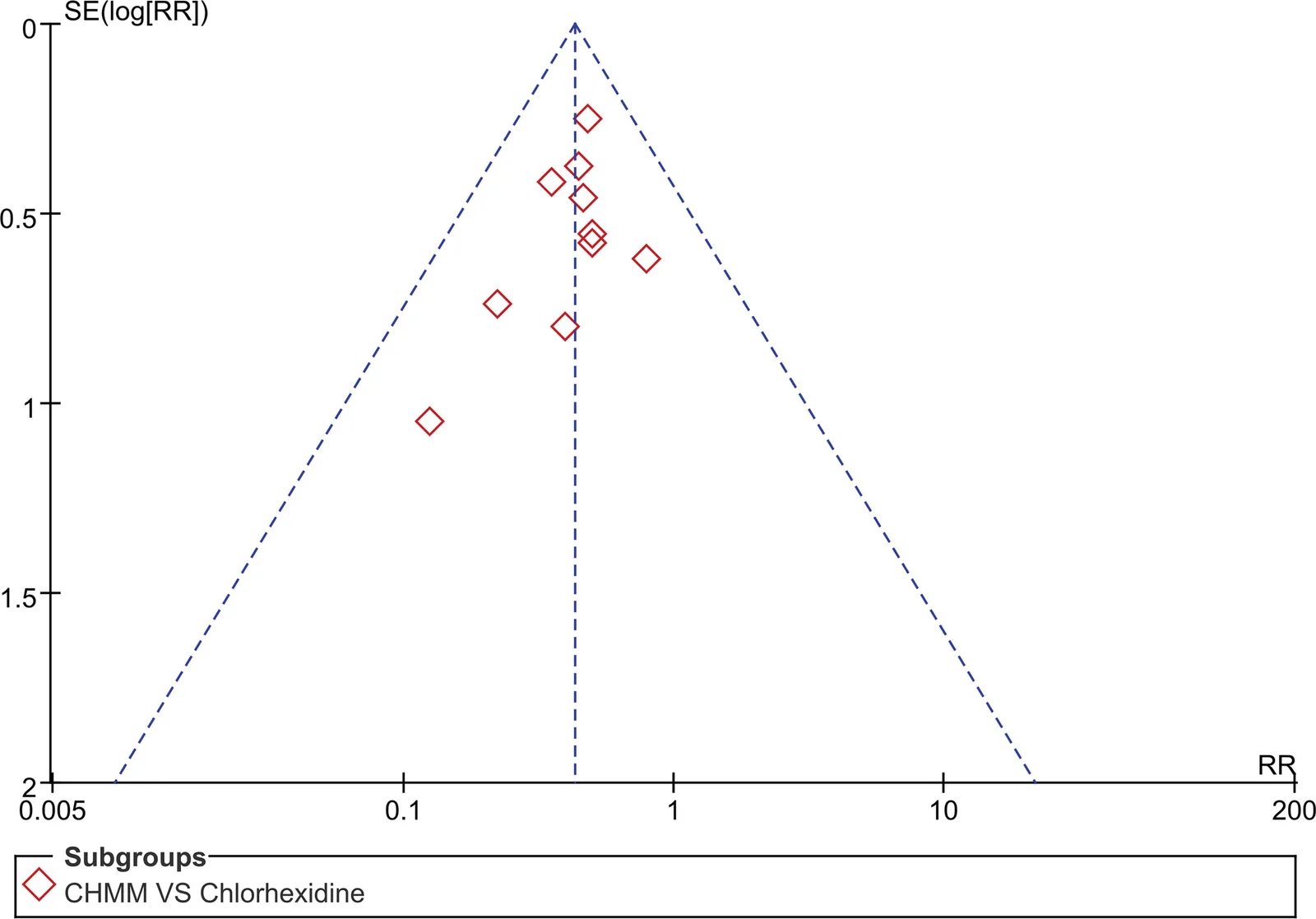
Funnel plot of the incidence of VAP in CHMM vs chlorhexidine. CHMM = Chinese herbal medicine mouthwash, RR = risk ratio, SE = standard error, VAP = ventilator-associated pneumonia.

We employed a funnel plot to explore the publication bias in the combined results. For studies using saline controls, the distribution of scatter points in the funnel plot (Fig. 13) exhibited asymmetry. And Egger's test indicated significant funnel plot asymmetry (intercept = −1.63, *P* = .007). Trim-and-fill analysis imputed 2 missing studies on the left side of the funnel, yielding an adjusted RR of 0.41 (95% CI: 0.30–0.56) compared to the original RR of 0.32. Trim-and-fill adjustment indicated an overestimation of the treatment benefits of CHMM efficacy. However, the adjusted result remains statistically significant, supporting the robustness of the primary conclusion. In chlorhexidine-controlled studies, visual inspection of the funnel plot (Fig. 14) revealed mild asymmetry, but Egger's test found no significant publication bias (intercept = −0.29, *P* = .735) and trim-and-fill analysis (0 imputed studies; adjusted RR = 0.43, 95% CI: 0.32–0.57) indicated no significant publication bias.

### 4.6. The certainty of the evidence

The summary of findings table for the main comparison between CHMM and non-CHMM was performed, which encompassed the outcomes of the incidence of VAP, oral ulcers, fungal infections, the duration of MV, and the score of the Modified Beck Oral Assessment Scale. The results showed that the certainty of evidence was rated moderate to very low (see Table 2).

**Table 2:** Summary of findings.

Comparison: CHMM VS Non-CHMM Population: patients with endotracheal intubation					
Outcomes	Anticipated absolute effects* (95% CI)		Relative effect (95% CI)	Number of participants (studies)	Certainty of the evidence (GRADE)
	Risk with non-CHMM	Risk with CHMM			
Incidence of VAP	266 per 1000	104 per 1000 (83–128)	RR 0.39 (0.31–0.48)	2187 (23 RCTs)	Low†^,^‡
CHMM vs saline	248 per 1000	74 per 1000 (50–107)	RR 0.30 (0.20–0.43)	902 (10 RCTs)	Moderate§
CHMM vs CHX	272 per 1000	111 per 1000 (81–152)	RR 0.41 (0.30–0.56)	928 (10 RCTs)	Low‡,‖
Incidence of oral ulcers	218 per 1000	39 per 1000 (30–50)	RR 0.18 (0.14–0.23)	1240 (15 RCTs)	Low‡,¶
Incidence of fungal infections	228 per 1000	71 per 1000 (48–107)	RR 0.31 (0.21–0.47)	806 (8 RCTs)	Low§,#
Duration of mechanical ventilation	–	SMD 2.1 lower (2.94 lower to 1.26 lower)	–	464 (5 RCTs)	Very low**,††
The score of the Modified Beck Oral Assessment Scale	–	SMD 2.2 lower (2.58 lower to 1.81 lower)	–	168 (1 RCT)	Low‡‡

GRADE Working Group grades of evidence.

High certainty: we are very confident that the true effect lies close to that of the estimate of the effect.

Moderate certainty: we are moderately confident in the effect estimate: the true effect is likely to be close to the estimate of the effect, but there is a possibility that it is substantially different.

Low certainty: our confidence in the effect estimate is limited: the true effect may be substantially different from the estimate of the effect.

Very low certainty: we have very little confidence in the effect estimate: the true effect is likely to be substantially different from the estimate of effect.

Explanations.

* The risk in the intervention group (and its 95% confidence interval) is based on the assumed risk in the comparison group and the relative effect of the intervention (and its 95% CI).

† Downgraded 1 level due to 8 studies at high risk of bias.

‡ Funnel plot is asymmetric.

§ Downgraded 1 level due to 3 studies at high risk of bias.

‖ Downgraded 1 level due to 2 studies at high risk of bias.

¶ Downgraded 1 level due to 5 studies at high risk of bias.

# . Downgraded 1 level due to only 8 studies reported on this outcome.

** Downgraded 1 level due to 1 study at high risk of bias.

†† Downgraded 2 levels due to only 5 studies reported on this outcome and substantial heterogeneity (*I*^2^ = 92%).

‡‡ Downgraded 2 levels due to very serious imprecision: only 1 study reported on this outcome, with data that did not enable us to evaluate the outcome.

CHMM = Chinese herbal medicine mouthwash, CHX = chlorhexidine, CI = confidence interval, RCT = randomized clinical trial, RR = risk ratio, SMD = standardized mean difference, VAP = ventilator - associated pneumonia.

## 5. Discussion

### 5.1. Principal findings

This systematic review included 27 RCTs with 2426 participants and demonstrated that CHMM or CHMM and routine oral care were more effective in preventing the incidence of VAP, oral ulcers, and fungal infections, shortening the duration of MV and reducing the score of the Modified Beck Oral Assessment scale, as compared with non-CHMM. However, we found the certainty of evidence was low or very low. In preventing the incidence of VAP, our findings were similar to Shan et al^[25]^ findings. In addition, our results also confirmed that CHMM may be superior to saline (low-certainty evidence) and chlorhexidine (moderate-certainty evidence) for preventing VAP. This result differed from the recommendation from the previous^[58]^ study. Mojtahedzadeh’s^[23]^ study only showed that CHMM also had similar and comparable effects to chlorhexidine on the prevention of lung infections in patients with MV, but did not address their potential priority.

In TCM, the core idea for understanding and treating diseases is syndrome differentiation and treatment and the holistic concept.^[59,60]^ TCM practitioners advocate for the selection of therapies and drugs based on the syndrome differentiation. VAP arises from the accumulation of pathogenic heat and dampness following the dysfunction of the oral cavity after ETT, according to TCM pathogenesis.^[49]^ Therefore, the herbs most often used in oral care are those that clear heat and remove dampness.^[49,61]^ For example, *lonicerae japonicae flos*, *menthae herba*, *licorice*, *scutellariae radix*, t*araxacum* and *forsythia,* which are recognized for their heat-clearing, dampness-eliminating, detoxifying, anti-inflammatory, and antibacterial effects.^[49,53,62,63]^ They can inhibit the formation and growth of bacteria,^[49,64]^ directly inactivate a variety of viruses,^[65]^ reduce complications of patients with ETT, and mitigate drug resistance and oral flora disruption. We thought that the fundamental concept of choosing therapies and medications according to syndrome differentiation may be a pivotal component in the enhanced effectiveness shown with CHMM. However, among the studies we included, only 1 study^[42]^ described the specific syndrome of VAP and selected CHMM based on the syndrome. Regrettably, the researchers failed to examine the prevalence of VAP across individuals with different syndromes. Furthermore, 2 included studies^[34,41]^ suggested that VAP should be categorized according to the beginning date of ETT. Early-onset VAP usually occurs within 4 to 5 days after ETT, whereas late-onset VAP is more likely to occur beyond 5 days.^[66,67]^ And Lin’s study^[34]^ showed that CHMM reduced the incidence of early-onset VAP. However, the effect was not obvious in the late-onset group. The reason for this result was that there were differences in oral flora in different VAPs.^[68]^ Li’s study^[41]^ also mentioned that VAP can be categorized based on the disease progression. However, Li’s study did not follow this categorization to conduct. We believe that the mismatch between CHMM and the kind of VAP may have led to an underestimation of CHMM’s effectiveness. So, we recommended that future researchers prioritize the classification of VAP and explore the best suitable CHMM for various types of VAPs. Moreover, it is important to explore the categorization link between TCM and Western medicine for VAP. Importantly, we recognized that variations in the specific CHMM formulations used across different studies represent a significant source of clinical heterogeneity. However, despite these differences in composition, all included CHMM formulations explicitly adhered to the core principles of heat-clearing, detoxifying, and dampness-resolving for preventing and treating VAP. This variation is an inherent feature of TCM practice. We could not conduct a comprehensive analysis of this source of heterogeneity.

In our review, the certainty of evidence was low. Beyond clinical heterogeneity in CHMM formulations, there were other confounding factors. For example, the frequency, duration, method and preparation of CHMM interventions and others. Although we performed subgroup analyses based on these factors as possible, inherent limitations in the available data precluded further subgroup analyses across other dimensions. Consequently, the findings of this review should be interpreted with caution.^[69–72]^ Furthermore, most studies we included did not provide detailed information on diagnostic criteria. For instance, none of the studies we included described the diagnostic criteria for oral ulcers and fungal infection, or how they conducted tests. But previous studies have shown that substantial controversy remains about the ideal sampling and microbiological techniques, and thresholds.^[72]^ Besides, many studies failed to report the diagnostic criteria and the method of VAP. Significantly, there is no accepted gold standard for the diagnosis of VAP. The weaknesses of traditional pneumonia surveillance definitions may limit their utility for measuring the impact of care improvement programs.^[73]^ Therefore, researchers need to explore more objective and definitive diagnostic criteria. We strongly recommend that researchers report the information about their research as much detail as possible in the future. This omitted information adversely impacted our further analysis and interpretation of the study’s results, limiting the extensive clinical application of CHMM.

Moreover, our funnel plot demonstrated asymmetric. Both Egger’s test and the trim-and-fill method revealed publication bias, which we attribute partially to the selective publication of these smaller trials that reported larger treatment effects^[74–77]^ Besides, other factors can contribute to small study effects, including selective reporting and clinical heterogeneity.^[78]^ These findings suggest that previous studies may have overestimated the beneficial effects of CHMM, a conclusion consistent with sensitivity analysis restricted to low-risk-of-bias studies. Nevertheless, the direction of treatment effects remained unchanged throughout. Lastly, in our systematic review, none of the research reported the adverse effects of CHMM. Consequently, we could not evaluate the safety profile of CHMM. This limitation may be attributable to insufficient monitoring durations and the impaired self-reporting capacity of intubated patients. Although these herbal components have been historically used in CHMM formulations, rigorous safety evaluation remains imperative. Future research must integrate robust safety endpoints to ensure the risk-benefit profile of CHMM is comprehensively evaluated before clinical implementation can be recommended.

## 6. Limitation

The main strengths of this study are the application of Cochrane methodology and adherence to PRISMA guidelines. Moreover, the results of the sensitivity analysis were optimistic, indicating that our meta-analysis results were robust. However, there are some limitations in our study. Firstly, the sample size of the included studies was small and the methodological quality was generally poor. The main reasons for this were errors in randomization methods and inadequate reporting. We encourage researchers to strictly adhere to the CONSORT statement, which helps readers to critically assess and interpret trial results.^[79]^ Besides, the certainty of evidence was low, which constrains the extensive clinical use of CHMM. The current evidence supports exploratory rather than routine clinical implementation, and CHMM should not replace evidence-based oral care protocols without further validation. Thirdly, due to the several types of CHMM included in our study, we could only infer that CHMM is useful in preventing VAP, but we were unable to distinguish which specific type of CHMM was the most effective. Furthermore, only 8 databases were searched for potential studies, which may have missed studies in other databases. Finally, we just included papers published in English and Chinese. Other potential studies published in other languages were excluded.

## 7. Conclusion

In conclusion, although our meta-analysis found that CHMM may demonstrate advantages over non-CHMM in reducing the incidence of VAP, oral ulcers and fungal infections, shortening mechanical ventilation duration, and improving oral health, we cannot definitively recommend its use at this time. First, the evidence base consists of studies with predominantly low-certainty evidence and substantial clinical heterogeneity. Moreover, the inherent complexity of multi-herbal TCM formulations is compounded by the prevalent use of non-standardized, institutionally prepared solutions across included trials. Importantly, no study reported the adverse events, rendering the safety profile of these CHMM uncertain. Therefore, we cannot recommend CHMM for routine clinical use at this time. Large and high-quality RCTs are also needed to definitively evaluate the safety and benefits of CHMM in the future.

## Author contributions

**Conceptualization:** Chunxiang Su.

**Data curation:** Yuntong Liu, Huihui Liu, Lu Ren, Zhangqi Li.

**Formal analysis:** Yuntong Liu, Huihui Liu, Lu Ren, Zhangqi Li.

**Investigation:** Yuntong Liu, Chunxiang Su.

**Methodology:** Shujin Yue, Xiaoda Li, Chunxiang Su.

**Supervision:** Shujin Yue, Xiaoda Li, Chunxiang Su.

**Visualization:** Yuntong Liu.

**Writing – original draft:** Yuntong Liu, Huihui Liu, Chunxiang Su.

**Writing – review & editing:** Yuntong Liu, Huihui Liu, Lu Ren, Shujin Yue, Zhangqi Li, Xiaoda Li, Chunxiang Su.

## Supplementary Material


