# Supplementary material for: Effectiveness of Chinese herbal medicine mouthwash in preventing ventilator-associated pneumonia in patients with endotracheal intubation: A systematic review and meta-analysis
Source: Medicine (Baltimore). 2026 Apr 10;105(15):e48179. doi: 10.1097/MD.0000000000048179 (PMC13593325; doi:10.1097/MD.0000000000048179)
Supplement: Supplementary file 1 [file medi-105-e48179-s001.pdf]

Graphical Abstract:

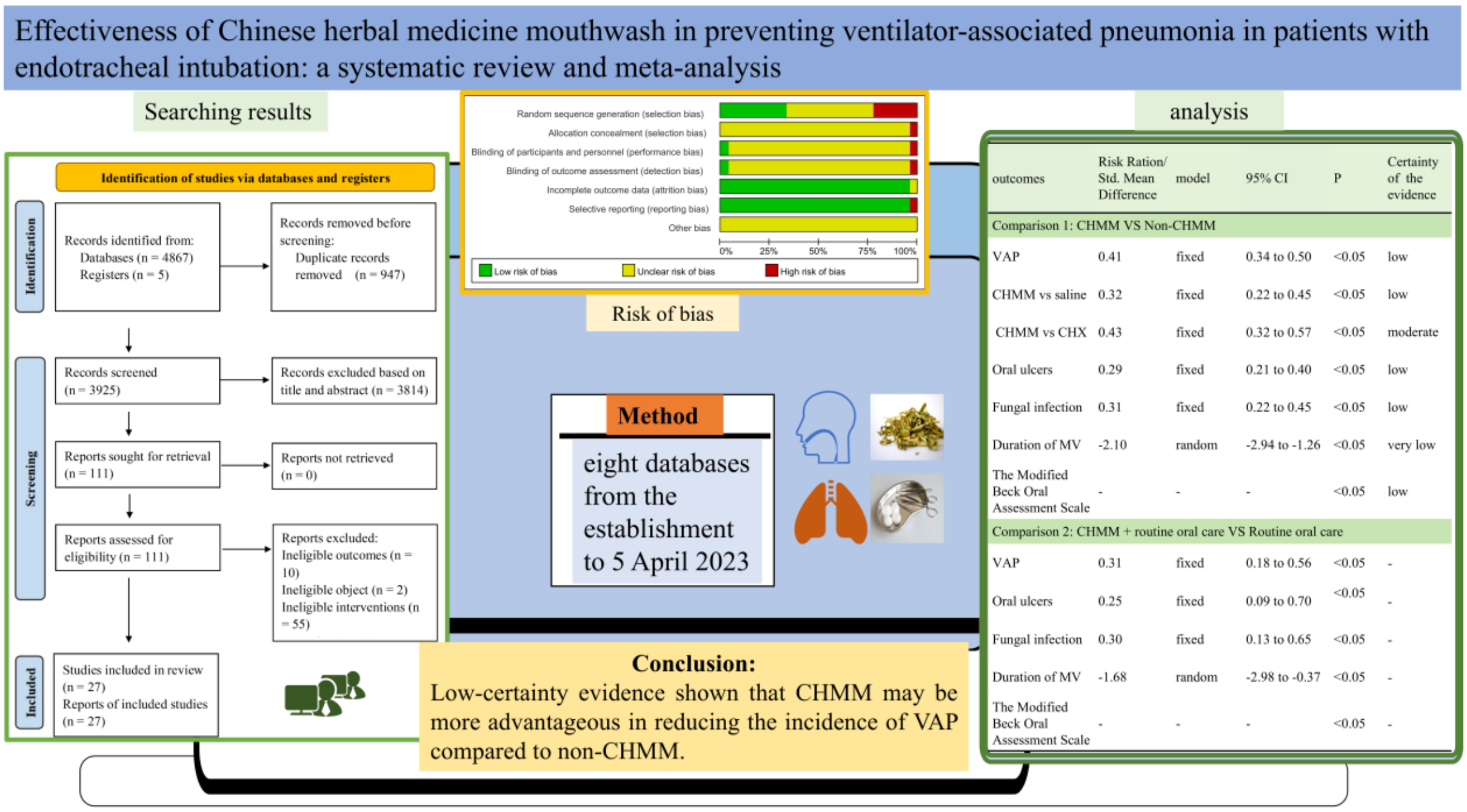

Supplementary file 1 Search strategy

Search strategy

CNKI Database search strategy

(((Title =(fuzzy match) "ventilator-associated pneumonia")) + ((Title =(fuzzy match) " VAP ")) + ((Title =(fuzzy match) "pneumonia")) + ((Title =(fuzzy match) "ventilator-related complications"))) * (((Title =(fuzzy match) "Chinese medicine")) + ((Title =(fuzzy match) "Chinese herbal medicine")) + ((Title =(fuzzy match) "Chinese herbal remedy")) + ((Title =(fuzzy match) "natural medicine"))) + (((Title =(fuzzy match) "oral care")) + ((Title =(fuzzy match) "oral rinse")) + ((Title =(fuzzy match) "oral hygiene"))+ ((Title =(fuzzy match) "oral health")) + ((Title =(fuzzy match) "rinse one's mouth"))+ ((Title =(fuzzy match) "gargle")))

Wan Fang Database search strategy

(((Topic =(fuzzy match) "ventilator-associated pneumonia")) OR ((Topic =(fuzzy match) " VAP ")) OR ((Topic =(fuzzy match) "pneumonia")) OR ((Topic =(fuzzy match) "ventilator-related complications"))) AND (((Topic =(fuzzy match) "Chinese medicine")) OR ((Topic =(fuzzy match) "Chinese herbal medicine")) OR ((Topic =(fuzzy match) "Chinese herbal remedy")) OR ((Topic =(fuzzy match) "natural medicine")) OR ((Topic =(fuzzy match) "oral care")) OR ((Topic =(fuzzy match) "oral rinse")) OR ((Topic =(fuzzy match) "oral hygiene")) OR ((Topic =(fuzzy match) "oral health")) OR ((Topic =(fuzzy match) "rinse one's mouth")) OR ((Topic =(fuzzy match) "gargle")))

VIP DATA search strategy

(((Title/KeywordTitle/Keyword =(fuzzy match) "ventilator-associated pneumonia")) OR ((Title/Keyword =(fuzzy match) " VAP ")) OR ((Title/Keyword =(fuzzy match) "pneumonia")) OR ((Title/Keyword =(fuzzy match) "ventilator-related complications"))) AND (((Title/Keyword =(fuzzy match) "Chinese medicine")) OR ((Title/Keyword =(fuzzy match) "Chinese herbal medicine")) OR ((Title/Keyword =(fuzzy match) "Chinese herbal remedy")) OR ((Title/Keyword =(fuzzy match) "natural medicine")) OR ((Title/Keyword =(fuzzy match) "oral care")) OR ((Title/Keyword =(fuzzy match) "oral rinse")) OR ((Title/Keyword =(fuzzy match) "oral hygiene")) OR ((Title/Keyword =(fuzzy match) "oral health")) OR ((Title/Keyword =(fuzzy match) "rinse one's mouth")) OR ((Title/Keyword =(fuzzy match) "gargle")))

Sinomed Database search strategy

("ventilator associated pneumonia"[all fields] OR "Ventilator-Associated Pneumonia"[all fields] OR "ventilator-associated pneumonia"[all fields] OR "pneumonia, Ventilator-related"[subject term]) AND "oral care"[common fields]

Pubmed Ovid search strategy

(("traditional Chinese herb"[Title/Abstract] OR "Chinese herb"[Title/Abstract] OR "Chinese medicine"[Title/Abstract] OR "Chinese herbal medicine"[Title/Abstract] OR "traditional Chinese medicine"[Title/Abstract] OR ("mouth wash*"[Title/Abstract] OR "mouth rins*"[Title/Abstract] OR "oral car*"[Title/Abstract] OR "gargle*"[Title/Abstract] OR "irrigat*"[Title/Abstract] OR "mist*"[Title/Abstract])) AND ("ventilator-associated pneumonia"[Title/Abstract] OR "pneumonia"[Title/Abstract] OR "VAP"[Title/Abstract] OR "ICU"[Title/Abstract])) AND (randomized controlled trial[Filter])

Embase Ovid search strategy

(“ventilator-associated pneumonia”:ti, ab, kw OR“pneumonia”:ti, ab, kw OR vap:ti, ab, kw OR icu:ti, ab, kw) AND (“traditional chinese herb”:ti, ab, kw OR “chinese herb”:ti, ab, kw OR “chinese medicine”:ti, ab, kw OR “chinese herbal medicine”:ti, ab, kw OR “traditional chinese medicine”:ti, ab, kw OR “mouth wash”:ti, ab, kw OR “mouth rins”:ti, ab, kw OR “oral car”:ti, ab, kw OR “gargle”:ti, ab, kw OR “irrigat”:ti, ab, kw OR “mist”:ti, ab, kw)

Cochrane Library search strategy

(("traditional Chinese herb"[Title/Abstract/Keyword] OR "Chinese herb"[Title/Abstract/Keyword] OR "Chinese medicine"[Title/Abstract/Keyword] OR "Chinese herbal medicine"[Title/Abstract/Keyword] OR "traditional Chinese medicine"[Title/Abstract/Keyword] OR ("mouth wash"[Title/Abstract/Keyword] OR "mouth rins"[Title/Abstract/Keyword] OR "oral car"[Title/Abstract/Keyword] OR "gargle"[Title/Abstract/Keyword] OR "irrigat"[Title/Abstract/Keyword] OR "mist"[Title/Abstract/Keyword])) AND ("ventilator-associated pneumonia"[Title/Abstract/Keyword] OR "pneumonia"[Title/Abstract/Keyword] OR "VAP"[Title/Abstract/Keyword] OR "ICU"[Title/Abstract/Keyword])) AND (trial[Filter])

CINAHL Ovid search strategy

(("ventilator-associatedpneumonia" [Title/Abstract/Keyword] OR "pneumonia" [Title/Abstract/Keyword] OR "VAP" [Title/Abstract/Keyword] OR "ICU"

[Title/Abstract/Keyword]))AND (("traditional Chinese herb"[subject terms] OR "Chinese herb"[subject terms] OR "Chinese medicine"[subject terms] OR "Chinese herbal medicine"[subject terms] OR "traditional Chinese medicine"[subject terms] OR ("mouth wash"[subject terms] OR "mouth rins"[subject terms] OR "oral car"[subject terms] OR "gargle"[subject terms] OR "irrigat"[subject terms] OR "mist"[subject terms]))

Supplementary file 2 Research data

Comparison 1: CHMM VS Non-CHMM				
Outcomes	CHMM		Non-CHMM	
	events	total	events	total
1.1 VAP				
1.1.1 CHMM VS Saline				
Dong 2017	4	20	8	20
He 2012	4	30	12	30
Li 2014	1	30	6	30
Li 2017	5	39	15	39
Li 2018	0	30	5	30
Liang 2020	4	82	16	82
Wu 2016	6	34	11	26
Yang 2019	5	40	13	40
Zhang 2021	5	120	14	120
Zhao 2011 A	2	30	11	30
1.1.2 CHMM VS Chlorhexidine				
Feng 2023	2	30	9	30
Fu 2021	8	50	18	50
Gao 2019	4	50	8	50
Gao 2020	6	60	13	60
Li 2019	1	60	8	60
Liao 2022	4	30	5	30
Lin 2022	4	30	8	30
Mojgan 2023	17	84	35	84
Xie 2022	6	40	17	40
Zhao 2011 B	2	30	5	30
1.1.3 CHMM VS hydrogen peroxide				
Lin 2014	22	67	33	72

Xu 2016	5	25	10	25
1.1.4 CHMM VS 0.1% povidone-iodine				
Xu 2021	1	50	8	50
1.1.5 CHMM VS Saline + chlorhexidine				
Zhang 2016	2	34	3	34
1.2 oral ulcers				
1.2.1 CHMM VS Saline				
He 2012	2	30	9	30
Li 2014	6	39	16	39
Li 2017	2	30	8	30
Liang 2020	5	82	14	82
Wu 2016	2	34	4	26
Zhao 2011 A	3	30	17	30
1.2.2 CHMM VS Chlorhexidine				
Feng 2023	0	30	4	30
Fu 2021	5	50	16	50
Gao 2019	3	50	12	50
Li 2019	2	60	10	60
Liao 2022	1	30	4	30
Lin 2022	3	30	5	30
Xie 2022	0	40	2	40
Zhao 2011 B	3	30	6	30
1.2.3 CHMM VS Saline + chlorhexidine				
Zhang 2016	0	34	3	34
1.2.4 CHMM VS hydrogen peroxide				
Xu 2016	5	25	15	25
1.3 fungal infection				
1.3.1 CHMM VS Saline				
Li 2017	5	39	15	39

Zhang 2021	4	120	12	120
Zhao 2011 A	3	30	15	30
1.3.2 CHMM VS Chlorhexidine				
Feng 2023	1	30	7	30
Fu 2021	10	50	23	50
Lin 2022	1	30	4	30
Xie 2022	4	40	13	40
Zhao 2011 B	3	30	6	30
1.3.3 CHMM VS Saline + chlorhexidine				
Zhang 2016	0	34	6	34

Comparison 1: CHMM VS Non-CHMM						
Outcomes	CHMM			Non-CHMM		
	Mean	SD	Total	Mean	SD	Total
1.4 Duration of MV						
1.4.1 CHMM VS Saline						
Feng 2023	12.52	1.24	30	16.4	1.58	30
Fu 2021	7.79	0.83	50	10.21	1.09	50
Lin 2022	105.374	42.352	30	143.876	79.001	30
Xie 2022	5.71	0.67	40	8.34	0.93	40
1.4.2 CHMM VS Chlorhexidine						
Liang 2020	6.87	1.54	82	9.46	1.63	82
1.5 Duration of MV						
1.5.1 CHMM VS Saline						
Liang 2020	6.42	1.02	84	8.95	1.26	84

Comparison 2: CHMM + Routine oral care VS Routine oral care				
Outcomes	CHMM + Routine oral care		Routine oral care	
	events	total	events	total

2.1 VAP				
Shi 2017	1	41	7	41
Wang 2020	4	30	10	30
Wu 2022	1	39	6	38
Yu 2021	6	25	15	25
2.2 oral ulcers				
Shi 2017	1	41	6	41
Wu 2022	3	39	10	38
2.3 fungal infection				
Shi 2017	0	41	4	41
Wu 2016	6	39	17	38

Comparison 2: CHMM + Routine oral care VS Routine oral care						
Outcomes	CHMM + Routine oral care			Routine oral care		
	Mean	SD	Total	Mean	SD	Total
2.4 Duration of MV						
Wang 2020	7.6	1.03	30	9.94	0.92	30
Wu 2022	9.31	1.75	39	11.31	2.08	38
2.5 Duration of MV						
Wang 2020	8.61	1.29	30	10.52	3.1	30
